# Cationic starch/pDNA nanocomplexes assembly and their nanostructure changes on gene transfection efficiency

**DOI:** 10.1038/s41598-017-14551-1

**Published:** 2017-11-01

**Authors:** Hongwei Wang, Xiaoxi Li, Ling Chen, Xiaoyi Huang, Lin Li

**Affiliations:** 1 Ministry of Education Engineering Research Center of Starch & Protein Processing, Guangdong Province Key Laboratory for Green Processing of Natural Products and Product Safety, South China University of Technology, Guangzhou, 510640 China; 2 Present Address: CAS Lamvac Biotech Co., Ltd., No.3, Lanyue Road, Guangzhou Science Park, Guangzhou Hi-Tech Industrial Development Zone, Guangzhou, Guangdong 510663 China

## Abstract

This study aims at developing biocompatible starch based gene carriers with good gene delivery and transfection efficacy. By controlling the molecular weight and aggregation behavior of spermine modified cationic starch (CS) molecules, nanocomplexes spontaneously formed through electrostatic interaction using CS and plasmid pAcGFP1-C1 (pDNA) displaying different structural changes (particle size, zeta potential, shape, compactness) response to the simulated intracellular pH variation. Results indicated that CS2 with weight average molecular weight (*Mw*) of 6.337 × 10^4^ g/mol displayed relatively higher transfection efficacy (~30%) in HepG2 cells than others and revealed significantly low cytotoxicity. By simulating the intracellular pH variation, Dynamic Light Scattering (DLS) and Small Angle X-ray Scattering (SAXS) results demonstrated that CS2 could bind to pDNA tightly and form nanocomplexes with smaller and compact internal aggregate structure at acidic conditions, which facilitated the effective pDNA protection under endosome pH change, while larger and loose internal aggregate structure at physiological pH which promoted the disintegration of CS2/pDNA nanocomplexes. Therefore, CS with suitable Mw of around 6.0 × 10^4^ g/mol represents a potential gene carrier for gene delivery. This study also demonstrated that controlling the internal nanostructure change of polymer/gene nanocomplexes could provide guidance in designing effective starch based gene carriers.

## Introduction

Being natural, renewable, nontoxic, biodegradable and biocompatible, cationic polysaccharides derived from renewable natural based resources have been regarded as the most attractive gene delivery carriers and are receiving more and more attention in gene transfection^1–7^. Recently, increasing consideration has been given to nano-scale particulate parameters including chemical properties, size, shape, surface charge and ligand modification. These parameters have been revealed to play a significant role in determining the extent and pathway of endocytic uptake, and the subsequent cellular trafficking of DNA to arrive intact within the nucleus to induce transgene expression^1,8–10^.

Starch is one of the most abundantly sustainable polysaccharides, basically derived from maize, potato, cassava, etc., with outstanding nontoxicity, good biocompatibility and biodegradability, consisting of two major components: amylose which is a primarily linear polysaccharide with α-(1-4)-linked D-glucose units and amylopectin which is a highly branched molecule, with α-(1-4)-linked D-glucose backbones and exhibits about 5% of α-(1-6)-linked branches^11^. A well accepted model defines the cluster structure of starch as a group of chains^11,12^. The external chains of starch interact with each other by hydrogen bonds and form helices structure that consists of a 9 nm repeat distance. Then these helices are organized into effective building ‘blocklets’ which range in diameter between 20 and 500 nm depending on starch botanical type and their location in the granule. Above all, the unique cluster structure of starch offers plenty binding sites for effective DNA condensation. In recent years, there have been studies targeted on starch in drug and gene delivery systems by applying appropriate chemical modification of starch molecule. Zhou *et al*. reported amylopectin with a *M*_w_ of 3.1 × 10^7^ g/mol as gene carriers by introducing various oligoamine residues to the hydroxyl groups present on amylopectin. The amylopectin derivatives were demonstrated to be biocompatible and could form nanoscale complexes lower than 300 nm with pDNA and exhibited high transfection efficiency in 293T cells^13^. Noga *et al*. reported the use of hydroxyethyl starch (HES) for the controlled shielding/deshielding of polyplexes in gene transfection by controlling the absence or presence of α-amylase (AA)^14^. Sieradzki *et al*.^15^ developed starch based gene delivery carriers by synthesizing cationically modified starch (Q-starch) with various origins and different molecular weight and nitrogen content by means of quaternization. Their studies showed that the complexation with pDNA, the particle size and zeta potential of the complexes were affected by the molecular weight, the nitrogen content of starch and the N/P ratios between starch and pDNA. They also demonstrated that there are two barriers for efficient transfection including the endosomal escape and complex de-complexation, and the latter appears to be the rate-limiting step. Although the above studies revealed that modified starch could be potential gene carriers, the relationship between the internal nanostructure of the complexes and their transfection efficiency has not been clearly elucidated. Research has shown that the transfection efficiency of the gene delivery systems is not completely determined by their particle size at normal physical pH value. In fact, whether the nanostructure of the nanocomplexes would change when they are internalized and trapped inside the endosome (experiencing pH changes from 7.4 to 5.0) could affect their DNA release profile and eventually the transfection efficiency, as demonstrated in one of our recent publications, wherein we had synthesized a series of cationic starch with different degree of substitution of spermine, a tetra-amine with two primary and two secondary amino groups that is involved in cellular metabolism and is present in all eukaryotic cells, and evaluated their transfection efficiency on HepG2 cells^16^. Recent studies also suggested that the introduction of spermine to natural polysaccharides like dextran, pullulan and chitosan have induced little cytotoxicity and enhanced the transfection efficiency^17–21^.

Previous research of our group has found that the unwinding or aggregation of external chains of starch molecules is greatly affected by the environmental changes (such as temperature, pH, moisture content etc), and the extent of changes is dependent on the chain length and various branched structure of starch molecules which results in different molecular weight of starch^22,23^. Based on these results, it is supposed that gene delivery systems formed by pDNA and starch with various molecular weight would experience different structure changes upon the cellular uptake of the nanocomplexes due to the cellular pH changes. Therefore, it would be significant to further the previous study by investigating how the molecular weight of starch affect the physicochemical properties of cationic starch (CS)/pDNA nanocomplexes and the transfection efficacy.

Thus, in this work, starch with different *M*_w_ ranging from 3.0 × 10^4^ g/mol to 1.5 × 10^5^ g/mol were obtained followed by the chemical modification with spermine. The cytotoxicity and transfection efficiency of the CS/pDNA nanocomplexes were investigated *in vitro*. The effect of *M*_w_ of starch molecules on the structural properties of CS/pDNA nanocomplexes including internal nanoscale structure, and particle size and zeta potential were analyzed by small angel X-ray scattering (SAXS) and dynamic light scattering (DLS) respectively, and the structural changes of CS/pDNA nanocomplexes under different pH conditions were correlated to the transfection efficiency. A schematic illustration of the relationship between the internal structural changes of CS/pDNA nanocomplexes and the transfection efficiency was presented.

## Results and Discussion

### Synthesis of CS and Structural Characterization

By controlling the extent of enzymatic hydrolysis of gelatinized starch by amylases, starch with various chain length and branched structure could be obtained. In this study, the gelatinized starch suspension was treated with thermostable α-amylase at different time and the spermine was introduced to maize starch hydrolysates with different *M*_w_ by the CDI activation method. The resulting products were labeled as CS1, CS2 and CS3 with the increase of *M*_w_. The *M*_w,_ *M*_w_ distribution, and root mean square radius of gyration of CS molecular chain(*R*_g_), the root mean square radius of gyration of the internal aggregates of CS(*r*_g_), and primary amine content of CS were shown in Table 1.

**Table 1:** Characterization of starch based carriers.

Samples	Cumulative weight fraction with different *M*_w_ range (%)^a^				*M*_w_(g/mol)^a^	NH_2_ content (μmol/mg)^c^	*R*_g_ (nm)^a^	*r*_g_^b^
	10^3^~10^4^	10^4^~10^5^	10^5^~10^6^	10^6^~10^7^				
CS1		99.12	0.84	—	3.826 × 10^4^	0.773	—	13.1
CS2	—	98.76	1.14	—	6.337 × 10^4^	0.967	13.7	5.2
CS3	—	0.73	98.99	—	1.468 × 10^5^	0.854	20.0	10.5

^a^ *M*_w_, *M*_w_ distribution, and *R*_g_ were determined by GPC-MALS. ^b^ *r*_g_ was determined by SAXS. ^c^NH_2_ content was determined by TNBS method.

CS with different *M*_w_ displayed similar primary amine content ranging from 0.7 to 1.0 μmol of NH_2_ per 1 mg of CS. The *M*_w_ of CS was determined to be 3.826 × 10^4^ g/mol, 6.337 × 10^4^ g/mol and 1.468 × 10^5^ g/mol respectively using the Gel Permeation Chromatography coupled with Multi-angle Light Scattering (GPC-MALS) system. As expected, the *R*_g_ of the CS molecular chain increased with increasing *M*_w_, and the *R*_g_ of CS1 was too small to be determined.

**Figure 1:**
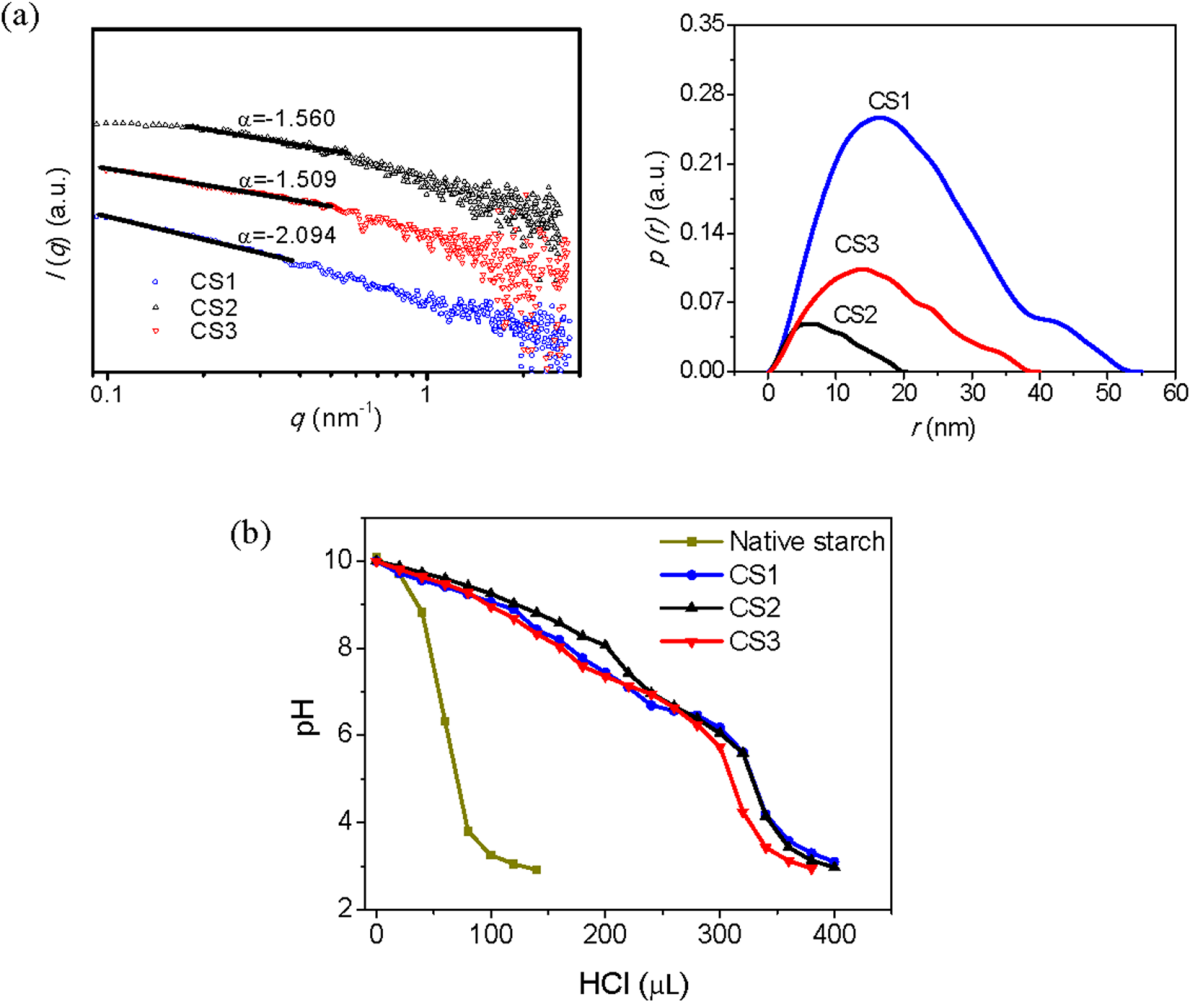
(**a**) SAXS analysis of nano-dimension of internal aggregate of CS (Left panel is the fractal properties and right panel is the *p(r)*~*r* curve) and (**b**) the buffering capacity of native starch and CS with different *M*_w_.

SAXS could be applied to analyze the size and the shape of the internal orderly aggregates of CS in nanoscale (1~100 nm). The *r*_*g*_ value of internal aggregate of CS molecules determined by the Guinier principle equation^24,25^, Ln [*I*(*q*)] = Ln [*I*(0)] − *r*_*g*_^2^ *q* ^2^/3 within the Guinier region (where *I* is the scattering intensity and *q* is the scattering vector), revealed that higher *M*_w_ of CS led to increased *r*_*g*_ (5~11 nm) except for CS1 (*M*_w_ < 6.337 × 10^4^ g/mol) which showed the highest value of *r*_*g*_ (~13 nm). This may due to the fact that CS1 molecules with shorter chain length aggregated more easily in terms of high concentration. Apart from that, the fractal characteristics determined by the power law *I* = *q* ^α^ (−4 < α < −1) can be used to describe the compactness of the internal orderly aggregates of CS through the slope α if there is linear relationship between log *I* and log *q* ^26^. In the case of −3 < *α* < −1, the scattering source is classified as a ‘mass fractal’. The relation between the mass fractal dimension *D*_m_ is defined as *D*_m_ = −*α*, which reveals the physical arrangement of the mass such as polymer segments. According to the fractal concept, higher *D*_m_ values indicate higher degree of compactness^26,27^. Results from Fig. 1a (left panel) showed that the log *I* versus log *q* graph of CS was linear with −3 < *α* < −1, indicating that the internal aggregates of CS could be defined as mass fractal and the fractal dimension *D*_m_ = −*α*. From the result, we can find that the *D*_m_ of CS decreased as the *M*_w_ of CS increased, indicating a decreasing compactness.

The structural properties of CS internal aggregates were also examined by a model-independent alternative way to analyze the SAXS curves (Fig. 1a (right panel)) using General Indirect Fourier Transformation (GIFT). This procedure results in a so-called Pair Distance Distribution Function (PDDF) *p(r)*, an essential real-space function that containing information about size, shape, and internal structure of the scattering^28^. For homogeneous particles, *p(r)* represents the histogram of distances between pairs of points within the particle. Obviously, its value is uniformly zero when *r* exceeds *r*_*max*_, the maximum dimension of the particle^29^. According to Mertens *et al*.^30^, globular particles display bell-shaped *p(r)* functions with a maximum at about *r*_*max*_/2. Elongated particles have skewed distributions with a clear maximum at small distances corresponding to the radius of the cross-section. Flattened particles display a rather broad maximum, also shifted to distances smaller than *r*_*max*_/2. A maximum shifted towards distances larger than *r*_*max*_/2 is usually indicative of a hollow particle. Particles consisting of well-separated subunits may display multiple maxima, the first corresponding to the intra-subunit distances, the others yielding separation between the subunits. The *p(r)* plots of CS revealed that except for CS1, *r*_max_ of CS became larger as *M*_w_ increased. The *p(r)* plots of CS1 displayed two peaks, suggesting that the internal aggregates of CS1 were dumbbell-shaped with two connected subunits, and the radius of the cross-section of the larger subunit was about 17 nm. The internal aggregates of CS2 and CS3 were like oblate ellipsoid and the radius of the cross-section of CS2 was around 5 nm while that of CS3 was about 15 nm.

Based on the SAXS results of CS, we suppose that CS with smaller *M*_w_ displayed a stronger aggregation tendency and formed more compact structures because of intermolecular interaction, while CS with larger *M*_w_ had less chance to aggregate due to relatively unfolded chain structures, which resulted in comparatively looser structures.

### Buffering Capacity

Acid−base titration was carried out to study the buffering capability of CS. It is well recognized that the high buffering capacity of cationic polymers substantially benefits their gene transfection efficiency due to the “proton sponge effect”^31^ which is similar to polyethylene imine (PEI), a cationic polymer carrying amounts of primary, secondary or tertiary amino groups, thus improving their absorbing capability toward hydrogen ion, and increasing the swelling of endosome to make it easier for the polymer/DNA nanocomplexes to escape into the cytoplasm^32^. The buffering capacity versus pH profile of native starch and CS with different *M*_w_ was compared as given in Fig. 1b. From the titration curve trend, it was found that all CS exhibit superior buffering capacity than native starch, namely less pH drop with the addition of the same amount of HCl, which could be ascribed to the presence of primary and secondary amino groups on CS. Research showed that the buffering capacity of PEI has much relationship with the *M*_w_ of PEI, mainly due to the fact that the backbone of PEI is made up of amino groups^33,34^. Therefore, the *M*_w_ changes would contribute to the changes of the density of amino groups which further affects the buffering capacity of PEI. Whereas in this research, starch with various molecular weight showed similar pH buffering profile mainly due to the fact that the D-glucose units of starch have significantly contributed to the *M*_w_ of starch molecules, which have little influence on the buffering capacity.

### Nanocomplex Formation of CS with pDNA

**Figure 2:**
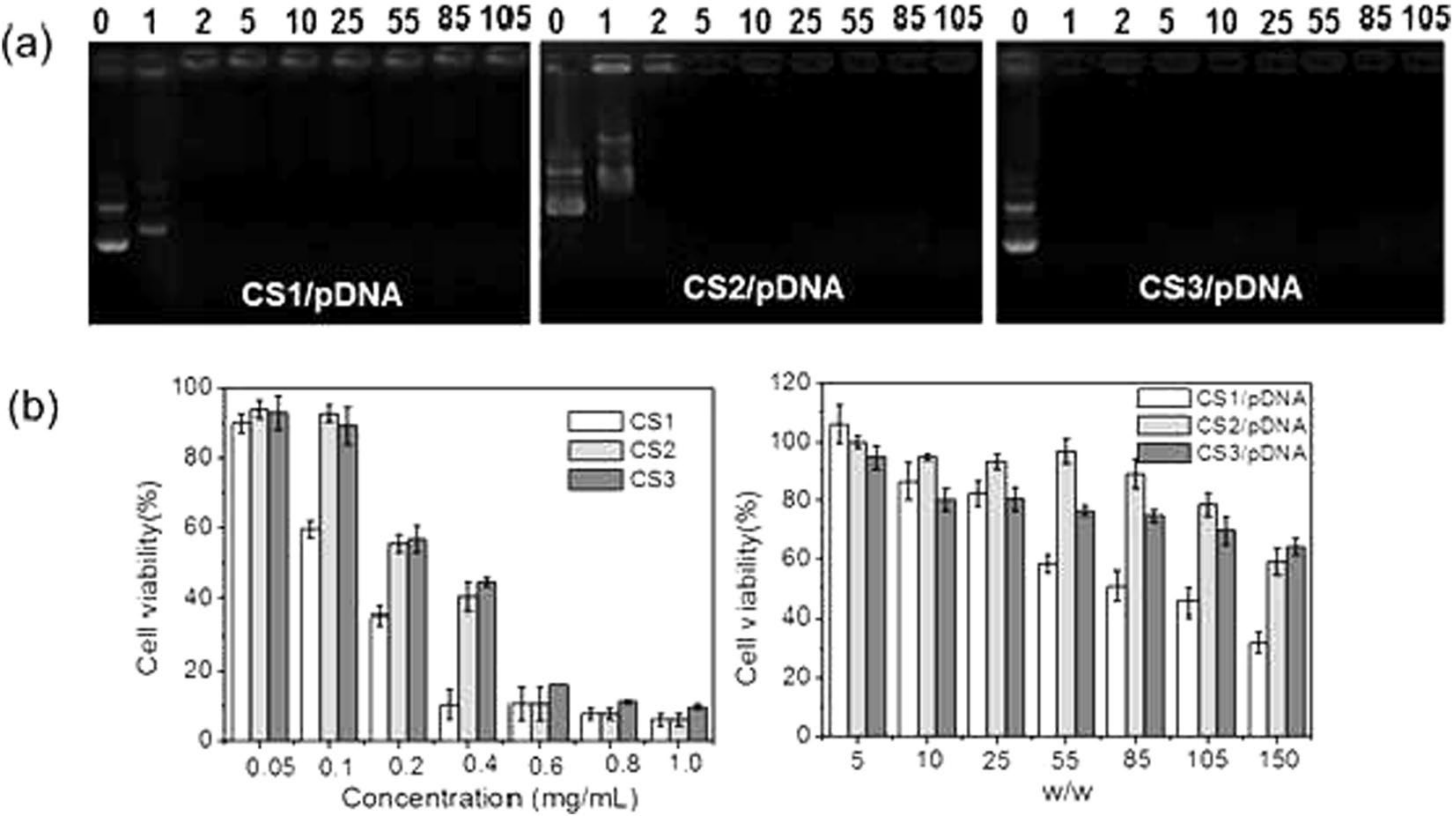
(**a**) Agarose gel electrophoresis retardation assay of CS/pDNA complexes at different weight ratios (from left to right is 0, 1, 2, 5, 10, 25, 55, 85 and 105, respectively) and (**b**) Cell viabilities of HepG2 cells in the presence of CS at various concentrations (left) and CS/pDNA complexes at different weight ratios (right). Data are shown as mean ± SD (n = 3).

In order to facilitate the uptake of CS/pDNA nanocomplexes to the negatively charged cell membrane, CS has to condense pDNA compactly and reduce the electrostatic repulsion between pDNA and cell surface by neutralizing the negative charge. In order to investigate the binding capability of CS to pDNA and determine the optimal complexation condition, the formation of nanocomplexes between pDNA and CS was observed by agarose gel electrophoresis (Fig. 2a). It showed that at the weight ratio of 1, CS3 was able to bind pDNA effectively whereas CS1 and CS2 failed to retard pDNA from migrating at this weight ratio even though CS3 displayed slightly lower primary amine content. Until at the weight ratio of 2, all the CS displayed complete retention of pDNA. Hence, the pDNA binding capacity of CS was mainly dependent on the *M*_w_ of CS. CS with higher *M*_w_ was more effective in binding pDNA and lower weight ratio between CS and pDNA was required for the complete condensation of pDNA. This could be attributed to existence of the chain entanglement effect of starch molecule through starch molecular aggregation and helix arrangement. CS with higher *M*_w_ (longer polymer chains and more branched structure) could entangle pDNA much more easily and compactly.

### Cell Cytotoxicity

The cell viability of CS with various concentrations ranging from 50 to 1000 μg/mL for HepG2 cells was determined by the MTT assay to assess their biocompatibility. As shown in Fig. 2b (left panel), it was found that the cell viability of cationic starch is relative to the concentration of the polymer. When the concentration was lower than 100 μg/mL, cell viability rate reached more than 90%. As the concentration went up, cell viability decreased substantially. Moreover, CS1 exhibited higher cell cytotoxicity toward HepG2 compared to other polymers even though the primary amine content was a little lower than that of CS2. This may be due to the fact that the positive charged amino groups of CS1 were more available to approach the negative charged cell surface because of its lower molecular weight and weaker steric hindrance.

The cytotoxicity of CS/pDNA nanocomplexes with various weight ratios (w/w = 5~150) were also evaluated as given in Fig. 2b (right panel). For CS/pDNA nanocomplexes formulated at different weight ratios, the CS concentration varied accordingly. The corresponding concentration of CS at increasing CS:pDNA (w/w = 5,10,25,55,85,105,150) was 0.01 mg/mL, 0.02 mg/mL, 0.05 mg/mL, 0.11 mg/mL, 0.17 mg/mL, 0.21 mg/mL and 0.3 mg/mL respectively. From Fig. 2b, we found that the cytotoxicity of CS/pDNA complexes was actually related to the toxicity of CS itself. When the weight ratios increased, the percentage of cell viability after incubated with CS/pDNA complexes was indeed in line with that after treated with CS alone at the same CS concentration, which suggested that the cytotoxicity of CS/pDNA was actually due to the toxicity of CS alone. The cell viability was higher than 80% at weight ratios lower than 55. And cell viability was still above 60% even when the weight ratio reaches 150 for all the nanocomplexes except for CS1/pDNA nanocomplexes, the cell viability of which was lower than 60% when weight ratios reached 55. It was thus indicated that CS could be biocompatible gene carriers and could find their potential application in cell transfection.

### Cell Transfection

**Figure 3:**
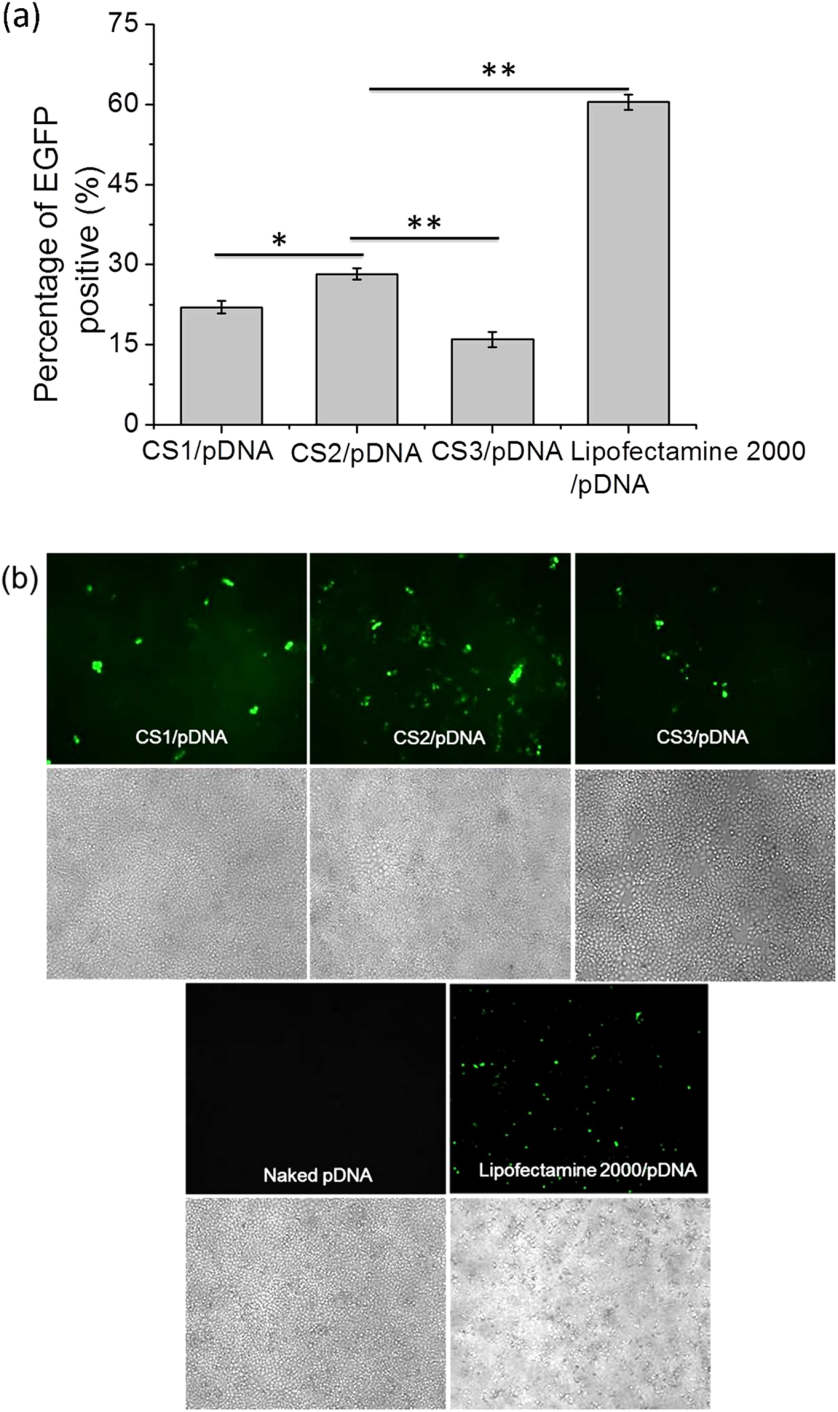
(**a**) Percentage of HepG2 cells expressing EGFP transfected by CS/pDNA complexes at the weight ratio of 55 after 48 h.; data was expressed in mean percentage of EGFP-positive population (n = 3, *p < 0.05, **p < 0.01, Students t-test) (**b**) Fluorescent images of EGFP expression in HepG2 cells after treatment with CS/pDNA complexes at the weight ratio of 55. Naked pDNA and lipofectamine 2000/pDNA were used as controls. The images were obtained by a fluorescent microscope at the magnification of 100× ; upper panel: fluorescent images; lower panel: bright field images.

The calculation of the actual percentage of EGFP-expressing cells quantitatively by analyzing the flow cytometry histograms (see Supplementary Fig. S1) is applicable to determine the gene transfection efficiency. As is shown in Fig. 3a, the gene transfection efficiency of CS/pDNA nanocomplexes at the weight ratio of 55 was related to the *M*_w_ of CS. Specifically, CS1/pDNA nanocomplexes had a gene transfection efficiency of 22% at the weight ratio of 55, and CS2 had superior transfection efficiency than CS1 with nearly 28% of cells expressing GFP. CS3 displayed the lowest transfection efficiency (~15%). As a positive control, lipofectamine 2000 displayed significantly higher transfection efficiency (~60%) than CS2. All the above results were in line with the results from fluorescence microscopy (displayed in Fig. 3b). From the bright field images we can also see that all the cells treated by CS/pDNA nanocomplexes were in good conditions suggesting low cytotoxicity. Although it revealed that percentage of cells expressing EGFP transfected by lipofectamine 2000/pDNA was comparatively high, the extreme cytotoxicity which was comparatively higher compared to all the CS/pDNA nanocomplexes would greatly compromise its value for effective gene transfection. Besides, it was obvious that pDNA was very weak in transfecting HepG2 cells.

### Morphology of CS/pDNA Nanocomplexes

**Figure 4:**
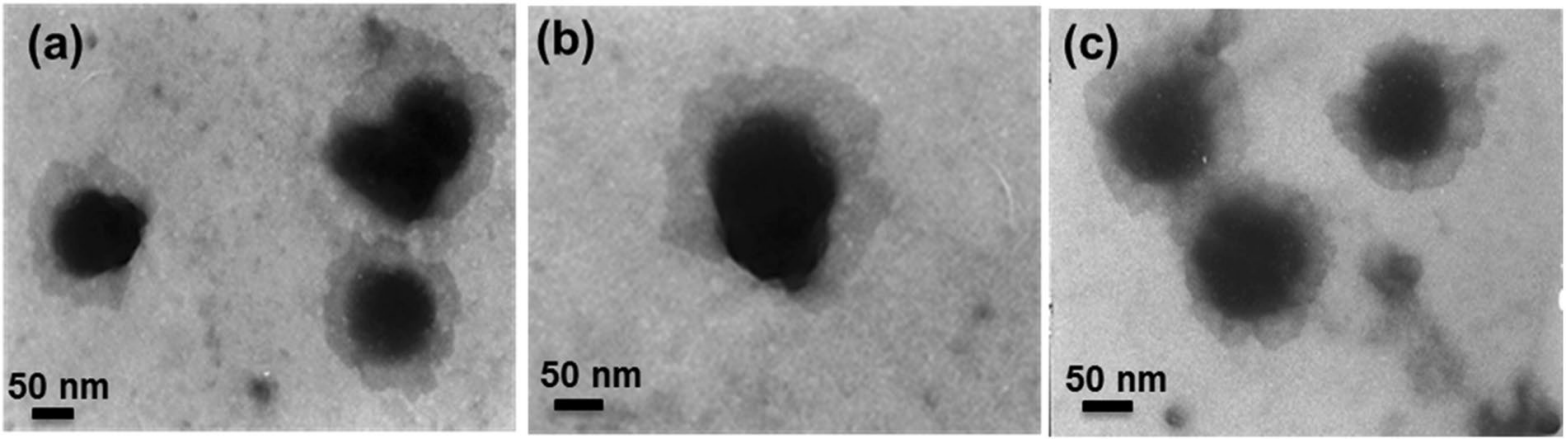
The TEM images of CSs/pDNA complexes at the weight ratio of 55. (**a**) CS1/pDNA; (**b**) CS2/pDNA; (**c**) CS3/pDNA.

The morphology of CS/pDNA nanocomplexes at the weight ratio of 55 was investigated by using transmission electron microscopy (TEM). Results (Fig. 4) showed that pDNA was well covered by starch layers and condensed effectively into nanocomplexes with sizes ranging from 100 to 200 nm. All the CS/pDNA nanocomplexes demonstrated globular or ellipsoidal morphologies with regular and compact structures.

### Effect of pH on the Structural Changes of CS/pDNA Nanocomplexes

Once internalized by HepG2 cells, the CS/pDNA nanocomplexes would be trapped inside the endosome in which pH rapidly drops to about 5 by the action of membrane bound ATP-driven proton pumps^35^. Our previous study had shown that the molecular weight of cationic starch had a great effect on the condensation of pDNA at different pH by using agarose gel electrophoresis. As displayed in Supplementary Fig. S2, CS/pDNA nanocomplexes prepared from cationic starch with smaller molecular weight (Mw = 1.792 × 10^4^) was quite sensitive to acidic environment when the CS/pDNA weight ratio was lower than 10. While for cationic starch with larger molecular weight (Mw = 5.527 × 10^4^), no pDNA leakage at both pHs was observed at all weight ratios. From the Supplementary Fig. S3, the results of the protection and release assay for CS/pDNA nanocomplexes prepared by cationic starch with different molecular weight showed that the pDNA-CS interaction is electrostatic driven and the interaction is pH sensitive. Therefore, the protection and release of pDNA by CS was greatly affected by the molecular weight of CS. However, the gene expression efficiency would also benefit from more complex reasons. The internal nano-structure of the CS/pDNA nanocomplexes could influence the pDNA release behavior across the CS aggregates. In this study, the effect of pH changes from 7.4 to 6.5 (simulating early endosome pH change) and 5.0 (simulating late endosome pH change) on the particle sizes and zeta potential as well as the internal structural changes of CS/pDNA nanocomplexes were investigated by DLS and SAXS respectively so as to evaluate the effect of *M*_w_ of CS on pDNA protection and delivery.

The hydrodynamic sizes and the zeta potentials of CS/pDNA nanocomplexes at different w/w ratios can be found as Supplementary Fig. S4 and the average size, zeta potential and polydispersity of the aggregates (PDI) changes of CS/pDNA nanocomplexes formed at w/w ratio of 55 under different pH conditions were shown in Table 2. The pH dependent size distribution histograms of each CS/pDNA nanocomplex were provided as Supplementary Fig. S5. From these DLS results we can see that at physiological pH (pH = 7.4), the size of CS/pDNA nanocomplexes were between 150 and 200 nm with a relative narrow size distribution, and the zeta potential ranged from 6 to 10 mV. Even though CS1/pDNA and CS3/pDNA displayed slightly higher positive surface charge than CS2/pDNA, their transfection efficiency was found to be lower than that of CS2/pDNA, which suggested that surface charge of the nanocomplexes was not the main reason for high transfection efficacy although higher positive charge was supposed to increase the cellular uptake of nanoparticles through electrostatic interaction with the cell membrane^36^. As pH decreased to 6.5 and 5.0, the average particle size of CS1/pDNA nanocomplexes increased significantly from 174 to 244 nm and 183 nm respectively, which suggests that CS1/pDNA nanocomplexes are quite vulnerable to acidic conditions and the structure became looser. The zeta potential of CS1/pDNA nanocomplexes decreased gradually as pH declined, which may be due to the partial exposure of the negatively charged pDNA molecules as a result of the loosened structure. The loosened structure of CS1/pDNA nanocomplexes would probably lead to the release of pDNA inside the endosome or into the cytoplasm after endosomal escape. On the contrary, the particle size of CS2/pDNA and CS3/pDNA nanocomplexes decreased slightly as pH dropped from 7.4 to 6.5 and 5.0, indicating more compact structure at acidic conditions. CS2 and CS3 demonstrated stronger molecular interaction with pDNA because of the intertwining effect originated from their larger molecular weight. Therefore, the exposure of pDNA was avoided and the zeta potential of these nanocomplexes increased as the amino groups on the CS chain became protonated when pH dropped to 6.5. However, as pH was decreased to 5.0, the further protonation of amino groups would probably increase the electrostatic repulsion between CS molecular chains to some extent, thus resulting in the exposure of pDNA and eventually the reduction of zeta potential. From the above results, we could conclude that the CS1 was less effective in protecting pDNA than CS2 at acidic conditions, so that the transfection efficiency of CS1/pDNA nanocomplexes was lower than that of CS2/pDNA nanocomplexes. It was difficult to explain the lower transfection of CS3/pDNA compared to that CS2/pDNA since both nanocomplexes displayed compact structures at acidic conditions according to the DLS results. Therefore, further internal structural analysis by SAXS was performed.

**Table 2:** The average size and zeta potential changes of CS/pDNA complexes formed at w/w ratio of 55 under different pH conditions.

Samples	pH	Average size (nm) w/w = 55	PDI	Zeta potential (mV)
	7.4	174.1 ± 3.1	0.272	9.72 ± 0.33
CS1/pDNA	6.5	244.3 ± 5.1*	0.187	7.77 ± 0.54*
	5.0	183.2 ± 5.7	0.279	7.38 ± 0.51*
	7.4	156.1 ± 5.3	0.321	6.18 ± 0.24
CS2/pDNA	6.5	153.1 ± 4.2	0.403	8.03 ± 0.27*
	5.0	144.6 ± 5.1	0.501	0.43 ± 0.10*
	7.4	185.6 ± 3.5	0.253	6.50 ± 0.41
CS3/pDNA	6.5	175.6 ± 4.7	0.424	8.00 ± 0.48
	5.0	163.5 ± 3.0	0.318	5.84 ± 0.26*

*P < 0.05. Data are shown as mean ± SD (n = 3).

**Figure 5:**
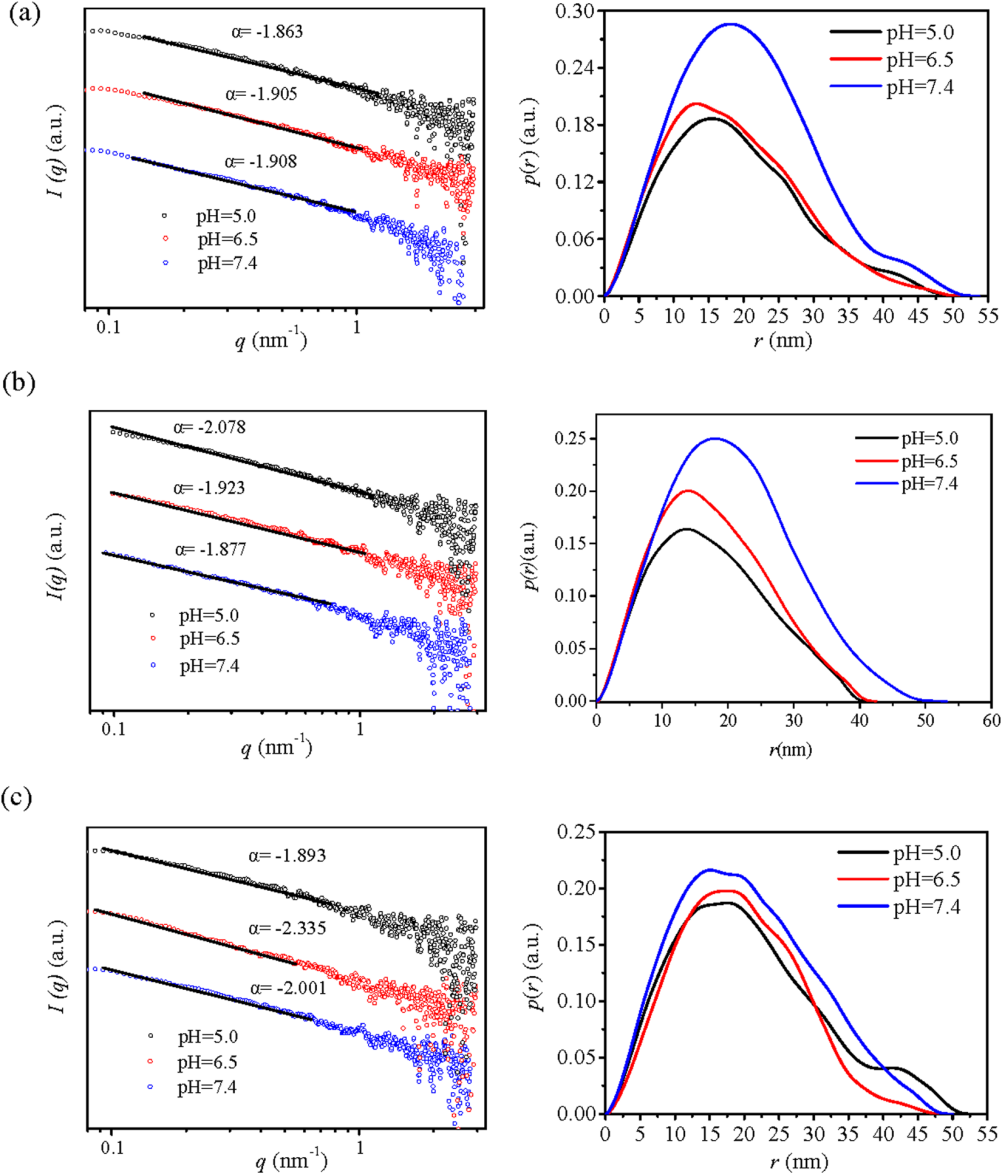
The fractal properties (left panel) and *p(r)*~*r* curve (right panel) of complexes formed by CS with different Mw and pDNA at a w/w ratio of 55 under different pH conditions. (**a**) CS1/pDNA; (**b**) CS2/pDNA; (**c**) CS3/pDNA.

The SAXS profiles of CS/pDNA nanocomplexes at different pH values showed that all the CS/pDNA nanocomplexes showed a featureless monotonic decay, revealing a disordered internal structure. The fractal characteristics from log *I* versus log *q* graph was used to describe the compactness of the internal orderly aggregates of CS/pDNA nanocomplexes. Results from Fig. 5 (left panel) showed that all the log *I* versus log *q* graph of CS/pDNA was linear and −3 < *α* < −1, indicating that the internal aggregates of CS/pDNA nanocomplexes could be defined as mass fractal and the fractal dimension *D*_m_ = −*α*. According to the *D*_m_ value of CS/pDNA nanocomplexes, the degree of compactness of CS/pDNA nanocomplexes was affected by pH. The *D*_m_ values of CS1/pDNA nanocomplexes decreased as pH declined from 7.4 to 6.5 and 5.0, indicating that the internal aggregates of CS1/pDNA nanocomplexes became looser within acidic environments, which was in line with DLS analysis. On the contrary, CS2/pDNA and CS3/pDNA nanocomplexes displayed much more compact internal structures since the *D*_m_ increased as pH reduced to 6.5. As pH further decreased to 5.0, the *D*_m_ of CS2/pDNA further increased displaying a comparatively stable structure at low pH with enhanced interaction between CS2 and pDNA; while for CS3/pDNA, the *D*_m_ decreased slightly, which suggested that the internal structure of CS3/pDNA became looser with weaker interaction between CS3 and pDNA, and this could be explained by the looser structure of CS3 with less aggregation tendency compared to CS2 according to previous discussion.

The *p(r)*-*r* profiles of CS/pDNA nanocomplexes under different pH indicated that the shape of the internal aggregates was strongly influenced by pH and the molecular weight of CS. As shown in Fig. 5 (right panel), the shape of CS1/pDNA aggregates changed from oblate ellipsoid with small subunits to prolate ellipsoid, and the maximum dimension *r*_max_ did not show significant changes (~50 nm) as pH decreased. While for CS2/pDNA nanocomplexes, the shape of the internal aggregates displayed typical profile of flattened prolate ellipsoid with a shrinking tendency of *r*_max_ (~50 nm → 40 nm) when pH changed from 7.4 to 6.5 and 5.0, which suggested that the internal structure became more compact. The shape of CS3/pDNA aggregates changed from oblate ellipsoid with multiple shoulders to prolate ellipsoid and then to oblate ellipsoid with a small subunit as pH dropped from 7.4 to 5.0, and *r*_max_ did not display significant changes (~50 nm).

According to the structure analysis by DLS and SAXS, the transfection efficiency of CS/pDNA nanocomplexes was mainly influenced by the interaction between CS and pDNA upon pH changes. Even though CS/pDNA nanocomplexes studied in this paper had suitable particle size and zeta potential for effective internalization by cells, they displayed different responsiveness (particle size, zeta potential, shape, compactness) towards pH changes thus leading to distinct transfection efficiency. CS1 with lower *M*_w_ of around 4.0 × 10^4^ g/mol is not so effective in intertwining with pDNA so that the structure of CS1/pDNA was sensitive to pH changes which could result in premature release of pDNA inside the endosome. CS2 with a higher *M*_w_ of around 6.0 × 10^4^ g/mol formed stable nanocomplexes with pDNA in acidic conditions which was beneficial in protecting pDNA from degradation in the endosomes and facilitating timely release of pDNA after escaping into the cytoplasm. Even though CS3 with *M*_w_ of around 1.5 × 10^5^ g/mol was capable of binding pDNA compactly close to neutral pH, the decreased compactness towards low pH because of its weaker aggregation tendency (according to SAXS results) may lead to lower transfection efficiency.

### The possible mechanism of internal structural changes of CS2/pDNA nanocomplexes

Apart from the electrostatic interaction between starch molecules and pDNA, possible hydrogen bonding between CS molecules may also exists. Besides, previous results of the SAXS analysis indicated that there were subunits present inside the CS/pDNA nanocomplexes. Therefore, it is supposed that the CS2/pDNA nanocomplexes consist of two parts, being the starch aggregates and the CS2/pDNA aggregates, which were connected by starch molecules.

**Figure 6:**
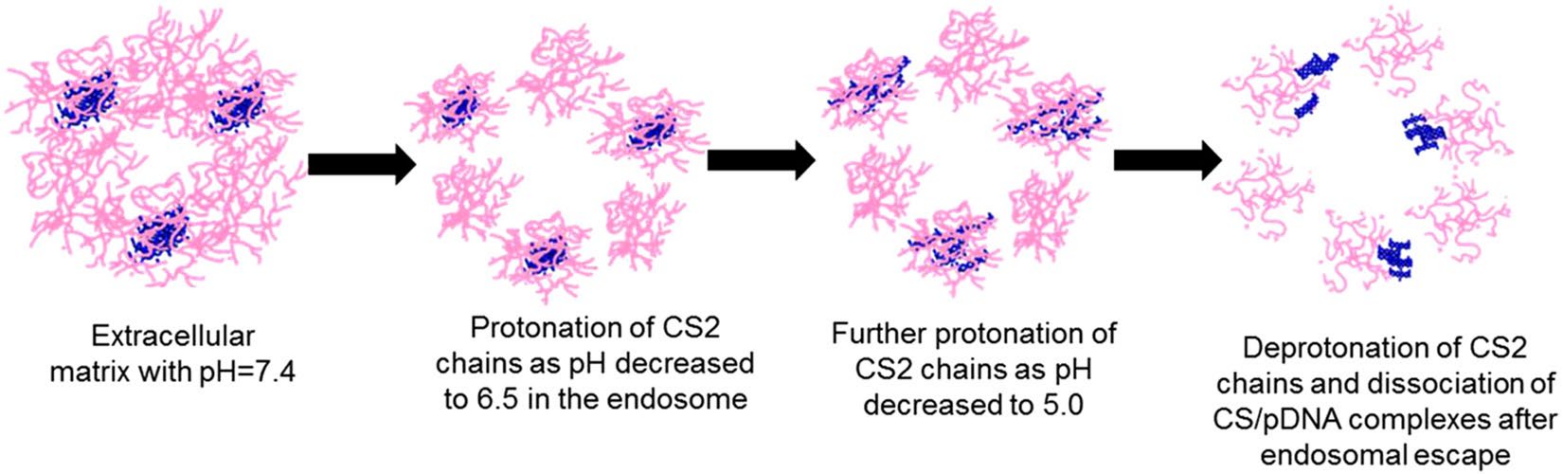
The possible schematic illustration of the internal structure changes of CS2/pDNA complexes during intracellular pH changes.

According to the analysis of the structural changes of CS2/pDNA nanocomplexes simulating the intracellular pH changes, the mechanism of intracellular gene trafficking was predicted as followed. As shown in Fig. 6, upon the cellular uptake by HepG2, CS2/pDNA nanocomplexes were trapped inside the early endosome, the slightly acidic condition of which led to the protonation of the amino groups of CS2, and caused the separation between starch aggregates and CS2/pDNA aggregates due to the electrostatic repulsion effect. As the early endosome further developed into the late endosome and lysosome, the further decrease of pH would increase the degree of protonation so as to the enhancement of the electrostatic repulsion between CS2 molecules, and consequently the partial exposure of pDNA. After escaping from endosomes, the deprotonation of CS2 resulted in the loose structure of the nanocomplexes and the final release of pDNA.

## Conclusion

Spermine modified cationic starch (CS) with *M*_w_ ranging from 3.0 × 10^4^ g/mol to 1.5 × 10^5^ g/mol were synthesized successfully and studied as gene vectors. CS exhibited superior pH buffering capacity compared to native starch. CS3 with *M*_w_ of around 1.5 × 10^5^ g/mol could condense pDNA effectively at lower weight ratio (w/w = 1) compared to CS with lower *M*_w_. *In vitro* transfection study in HepG2 cells showed that CS2 with *M*_w_ of 6.337 × 10^4^ g/mol displayed relatively higher transfection efficacy than others and revealed significantly low cytotoxicity. DLS and SAXS results demonstrated that CS2 could bind to pDNA compactly and form nanocomplexes with extremely higher stability at acidic conditions, thus leading to better transfection efficacy than CS1/pDNA and CS1/pDNA. This study indicated that being biocompatible and presenting good transfection efficacy, CS with suitable molecular weight of around 6.0 × 10^4^ g/mol represents a potential gene vector for gene delivery. Moreover, this study has demonstrated that the internal structure changes of polymer/gene polyplexes provides a new insight in designing effective polymer based gene vectors.

## Materials and Methods

### Materials

Maize starch with a *M*_w_ of 3.788 × 10^7^ g/mol was acquired from Huanglong Food Industry Co., Ltd. (Changchun, China). Thermostable a-amylase (AA) was bought from Novozymes Co., Ltd. (Bagevaerd, Denmark). Dimethyl sulphoxide (DMSO, chromatography degree) was obtained from Honeywell Burdick & Jackson (USA). N,N′-Carbonyldiimidazole (CDI) and spermine were purchased from Aladdin Reagent Company (Shanghai, China). Fetal bovine serum (FBS), RPMI-1640, penicillin-streptomycin, trypsin and PBS (pH = 7.4) were purchased from Hyclone Co. (Carlsbad, CA, USA). Methylthiazolyldiphenyl-tetrazolium bromide (MTT) and 2,4,6-trinitrobenzenesulfonic acid sol (TNBS) were obtained from Sigma Chemical Co. (St. Louis, MO, USA). Lipofectamine 2000 reagent was purchased from Invitrogen (Carlsbad, CA, USA). The plasmid pAcGFP1-C1 (4.7 kb; Clontech, Palo Alto, CA, USA) encoding enhanced green fluorescent protein (EGFP) was maintained and propagated in DH5αstrain of *E.coli*, and then purified by using the Endfree plasmid kit (Tiangen, China). The purity and concentration of plasmids were determined by UV spectrophotometry (A260/A280). All other reagents used in this experiment were of analytical grade and used without further treatment.

### Degradation of Maize Starch with Thermostable α-amylase and Characterization

Maize starch (15%, w/v) was pre-gelatinized by stirring in distilled water for 20 min at 100 °C. The starch paste was treated with a thermostable α-amylase with pH 6.0 and the mixture was incubated in a shaking boiling water bath for various reaction times (8 h, 12 h and 24 h respectively). After inactivating the enzymes, the products were dried at 65 °C, smashed and sieved to sizes of 80 mesh.

The *M*_w_ of the samples was measured by gel permeation chromatography (GPC) coupled with 18 angle multi-angle light scattering (MALS) detector (DAWN HELEOS MALS, Wyatt Technology Corp.) with a laser of wavelength 658 nm and a refractive index (RI) detector (Optilab rEx, Wyatt Technology Corp.). Samples were prepared at a concentration of 5 mg/mL using the mobile phase (DMSO containing LiBr (50 mM)) and filtered using a membrane filter (PTFE 5.0 mm, Millipore Corporation, Bedford, MA). The samples were injected (500 μL) into a Waters Styragel HMW 6E column (7.8 × 300 mm) and the flow rate of the system was set at 0.5 mL/min.

### Preparation and Structural Characterization of CS

#### Preparation of CS

CS was prepared through the introduction of spermine to the hydroxyl groups of maize starch by using the CDI activation method^37^. Briefly, maize starch with different *M*_w_ (1.0 g) was dissolved in 50 mL of anhydrous DMSO, and then CDI (250 mg) was added and stirred for 2 h at room temperature followed by the addition of spermine (312 mg). After the reaction was performed at 35 °C for 24 h, the reaction mixture was dialyzed against distilled water with a dialysis membrane (molecular weight cutoff 7,000), and then lyophilized to obtain CS. The primary amine content of CS was measured by the standard protocol of the TNBS method^38^. In brief, 20 μL of freshly prepared aqueous TNBS solution (15 mg/mL) was separately added to marked tubes containing up to 0.2 μmol spermine dissolved in 600 μL of deuterium depleted water (DDW). The mixtures were separately diluted with 200 μL of sodium bicarbonate buffer (0.1 M), vortexed for 1 min, and incubated for 2 h at 37 °C. Then, 450 μL of 1 N HCl aqueous solution was added to each tube, vortexed for 1 min, and gently sonicated for 2 min to remove bubbles. Absorbances of samples were recorded at 410 nm. A sample containing the same composition (without spermine) was used as a reference in the absorbance measurements. CS (1 mg/mL) was treated as above, and the primary amine content was calculated according to the calibration curve.

### GPC-MALS Analysis

The *M*_w_ of the prepared CSs was measured by GPC-MALS with Waters Shodex OH-804 columns. Samples were prepared at a concentration of 5 mg/mL with filtered mobile phase (0.1 M NaNO_3_ solution with 0.02% w/v NaN_3_ which had been filtered through 0.22 μm cellulose nitrate filter). The flow rate of the system was set at 0.5 mL/min.

### SAXS Analysis

The internal aggregate structure of CS was analyzed by SAXS (Anton-Paar, Graz, Austria). A PW3830 X-ray generator with a long fine focus sealed glass X-ray tube (PANalytical) was operated at 40 kV and 50 mA. A focusing multilayer optics and a block collimator provide an intense monochromatic primary beam (Cu-Kα, *λ* = 0.1542 nm). A semi-transparent beam stop enables measurement of attenuated primary beam at zero scattering vector. Aqueous suspensions of CSs around 10 mg/mL were directly filled into a capillary of 1 mm diameter and 0.01 mm wall thickness. The sample-to-detector distance was 261.2 mm, and the temperature was kept at 26.0 °C. The intensity profile was output as the plot of the scattering intensity (*I*) vs. the scattering vector, *q* = 4π/*λ*sin(*θ*/2) (*θ* = scattering angle). Each measurement was collected for 45 min. All *I*(*q*) data were normalized so as to have the uniform primary intensity at *q* = 0 for transmission calibration. The background scattering contributions from capillary and solvent were corrected. Desmearing is necessary because of the line collimation. The SAXS curves were analyzed using General Indirect Fourier Transformation (GIFT) to confirm the structural information of CS. This procedure results in a so-called Pair Distance Distribution Function (PDDF) *p(r)*, an essential real-space function that containing information about size, shape, and internal structure of the scattering^28^.

### Buffer Capacity Analysis

The buffer capacity of CS with different *M*_w_ was determined by acid-base titration in accordance with the method of literature^39,40^. Briefly, 0.2 mg/mL of each sample solution was prepared in 30 mL of 150 mM NaCl solution. The sample solution was first titrated by 0.1 M NaOH to a pH of 10, and then 0.1 M HCl solution with particular volume was added to the solution to obtained mixtures with different pH values, which were determined using a microprocessor pH meter (PB-10, Sartorius, Germany).

### Preparation of CS/pDNA Nanocomplexes

CS was dissolved in phosphate buffer solution (PBS, pH 7.4) with a concentration of 2 mg/mL and then filtered through 0.22 μm cellulose nitrate membrane filters. 5 μL of pDNA solution (200 ng/μL) was then added to the CS solution at different CS/pDNA weight ratios (w/w), followed by gentle agitation for 30 s and then incubation for 30 min at room temperature for spontaneous nanocomplex formation through electrostatic interaction between CS and pDNA.

### DNA Condensation Assays

The pDNA binding abilities of maize starch and CS were evaluated by agarose gel electrophoresis. The nanocomplexes prepared at various w/w ratios ranging from 1–105 were loaded into individual wells of 1.0% agarose gel in Tris-acetate (TAE), electrophoresed at 100 V for 30 min, and stained with ethidium bromide at 0.1 μg/mL. The resulting pDNA retardation patterns were observed under UV irradiation using a Bio-Rad electrophoresis system (Bio-Rad Laboratories, CA, USA).

### Characterization of CS/pDNA Nanocomplexes

#### DLS Analysis

Particle sizes and zeta potential of CS/pDNA nanocomplexes were measured in PBS buffer (10 mM, pH = 7.4) using Zetasizer Nano ZS (Malvern Instruments Ltd., Worcestershire, UK) at 25 °C. Each measurement was performed in triplicate.

#### Morphology Analysis

The morphologies of the different nanocomplexes were analyzed using transmission electron microscopy (TEM). The samples were prepared by placing a drop of nanocomplexes (CS/pDNA ratio (w/w) of 55 with pDNA concentration of 100 ng/μL) on a copper grid and stained with 1% uranyl acetate solution for 5 seconds. The grid was allowed to dry further for 10 min and examined with a JEM-1010 transmission electronmicroscope operated at 100 kV.

#### SAXS Analysis

The internal aggregate structure of CS/pDNA nanocomplexes was analyzed by SAXS as previously described.

#### Cytotoxicity Assay

The cytotoxicity of CS/pDNA nanocomplexes was evaluated by the MTT assay. HepG2 (human hepatoma cell line, China Center for Type Culture Collection, CCTCC-GDC024) cells were seeded in 96-well plates at a density of 1 × 10^4^ cells/well in100 μL RPMI 1640 containing 10% FBS and cultured at 37 °C for 24 h. After the medium was replaced by fresh serum-free medium, solutions of CS with different concentrations or CS/pDNA nanocomplexes (containing 0.2 μg pDNA) with different w/w were added to the culture medium of each well to assess their cytotoxicity. After incubation of the treated cells for another 24 h, 20 μL of MTT (5 mg/mL in PBS) was added to each well. After 4 h, the medium was carefully discarded from each well, and 150 μL DMSO was added to dissolve the MTT formazan crystals. The plate was incubated for another 10 min to have the MTT formazan crystals completely dissolved. Afterwards, the optical density was performed using an ELISA microplate reader (Muliskan Go, Thermo Fisher Scientific Inc.) at 490 nm. Each experiment was done in triplicate. Non-treated cell (in PBS) was used as a control and the relative cell viability was expressed as follows: Cell viability (%) = A_test_/A_control_ × 100.

#### Cell Transfection

Cells from HepG2 cell lines were plated in 24-well plates with 5 × 10^4^ cells/well in 500 μL RPMI 1640 containing 10% FBS and cultured at 37 °C for 24 h. The medium in each well was then replaced with fresh complete medium and the CS/pDNA nanocomplexes (containing 1 μg of pDNA) at different w/w were added. After incubation for 4 h, the transfection medium was removed and replaced by fresh medium containing 10% FBS. After further incubation for 48 h, cells were directly observed on a fluorescence microscope (Axiovert 200, Zeiss Germany). Subsequently, the transfected cells were washed twice with PBS (pH 7.4) and detached with 0.05% trypsin. FACS Aria flow cytometer (Guava EasyCyte, Meck Millipore, USA) was used to evaluate the gene transfection efficiency by calculating the percentage of cells expressing green fluorescence protein (GFP). Fluorescence parameters from 5,000 cells were acquired, and transfection was carried out in triplicate. The lipofectamine 2000/pDNA nanocomplexes were used as the positive control and naked pDNA used as the negative control.

### Structure Changes in CS/pDNA Nanocomplexes during the Simulated Intracellular pH Changes

Structure changes including particle size, zeta potential changes as well as the internal aggregate structure of CS/pDNA nanocomplexes were investigated by Zetasizer Nano ZS and SAXS respectively as the pH of the aqueous solutions dropped down from 7.4 to 6.5 and 5.0, simulating the pH changes when the CS/pDNA nanocomplexes were internalized into cells by endocytosis.

### Statistical Analysis

The data were presented as mean ± standard deviation (SD) of three different replicates and analyzed for statistical significance by Student’s two-tailed t-test (assuming equal variance), and a value of *P* < 0.05 or *P* < 0.01 was considered statistically significant.

### Data availability

All data generated or analysed during this study are included in this manuscript.

## Electronic supplementary material

Supplementary Information
