# Supplementary material for: Cationic starch/pDNA nanocomplexes assembly and their nanostructure changes on gene transfection efficiency
Source: Sci Rep. 2017 Nov 1;7:14844. doi: 10.1038/s41598-017-14551-1 (PMC5665959; doi:10.1038/s41598-017-14551-1)
Supplement: Supplementary file 1 — Supplementary Information [file 41598_2017_14551_MOESM1_ESM.doc]

Cationic starch/pDNA nanocomplexes assembly and their nanostructure changes on gene transfection efficiency

Hongwei Wang, Xiaoxi Li*, Ling Chen, Xiaoyi Huang^a^*, Lin Li

Ministry of Education Engineering Research Center of Starch & Protein Processing, Guangdong Province Key Laboratory for Green Processing of Natural Products and Product Safety, South China University of Technology, Guangzhou 510640, China

^*^ Corresponding authors. Tel.: +86 20 8711 3252; fax: +86 20 8711 3252. *E-mail addresses:* xxlee@scut.edu.cn (X. Li); hxy198707@126.com (X. Huang)

^a^ Present address of Dr. Xiaoyi Huang: Department of Chemistry, Materials, and Chemical Engineering “G.Natta”, Politecnico di Milano, Milan 20131, Italy


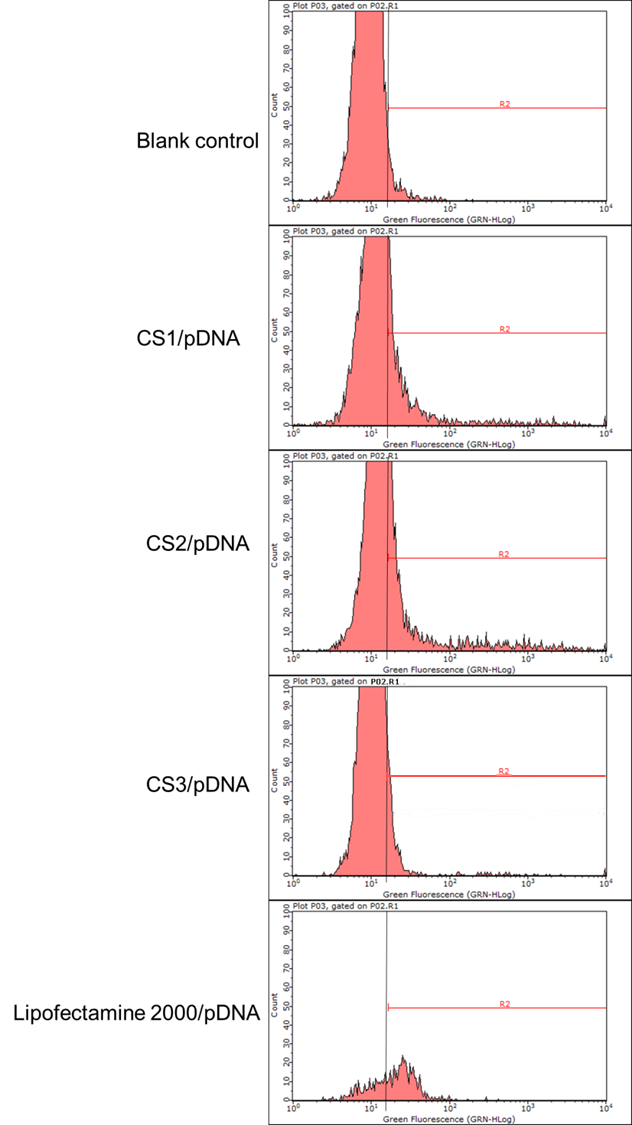


Fig. S1. Flow histograms of transfected HepG2 cells after exposure to CS/pDNA. PBS was used as the blank control while lipofectamine 2000 was used as the positive control.


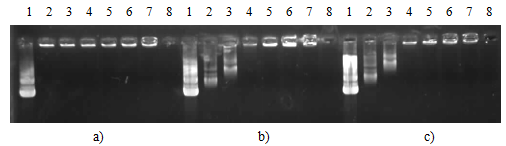


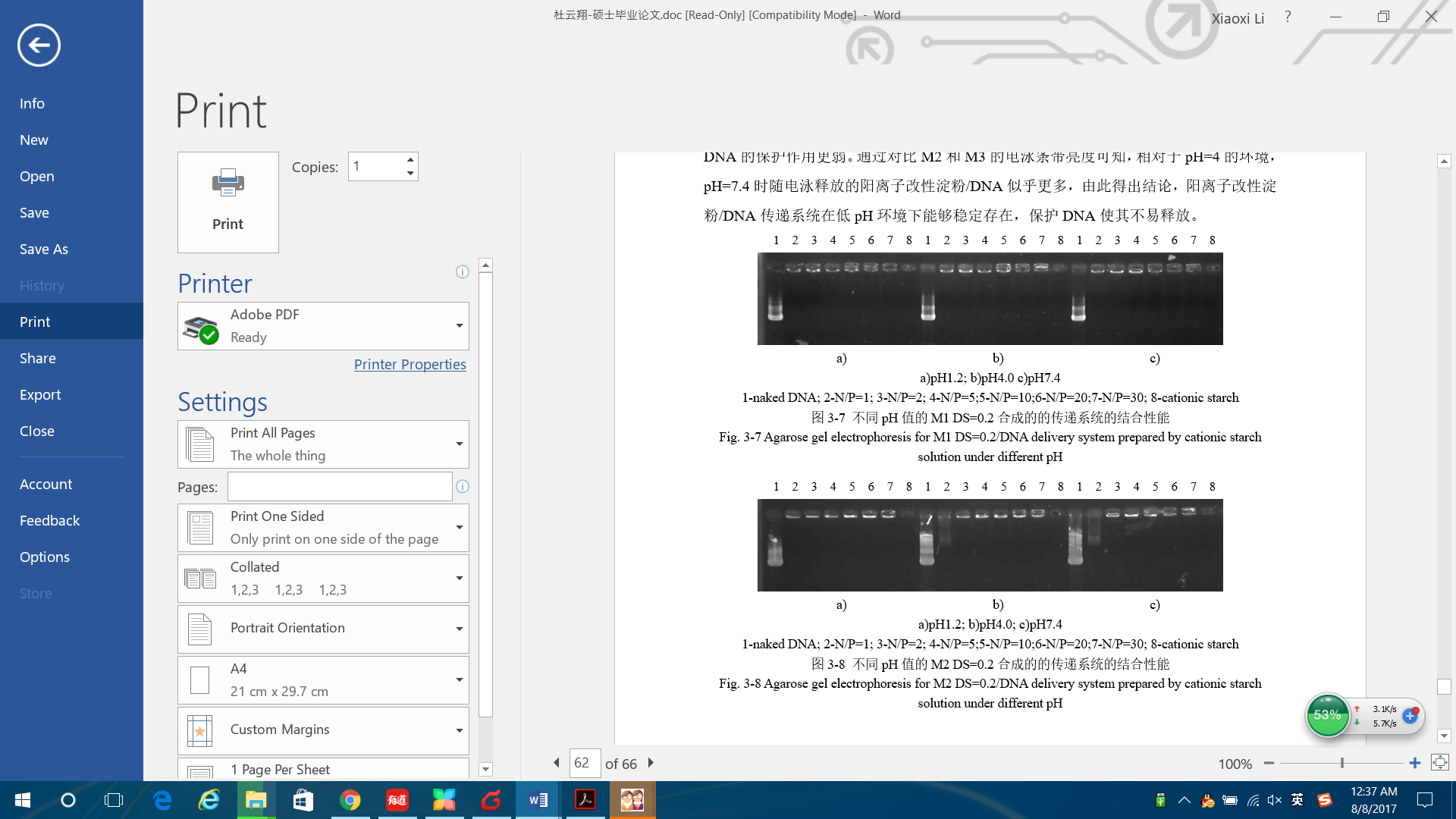


(a) (b)

Fig. S2. Agarose gel electrophoresis retardation assay for CS/pDNA complexes prepared by cationic starch with different molecular weight (the up Mw is 1.792×10^4^ and the bottom is 5.527×10^4^) at different weight ratios (from left to right is naked DNA, 3, 6, 10, 20, 40, 60 and cationic starch respectively) under different pH ((a) pH4.0; (b)pH7.4)

**1 2 3 4 5 6 7 1 2 3 4 5 6 7**

**Lysozyme - + + + + + + - + + + + + +**

**Heparin sodium - + + + + + + - + + + + + +**


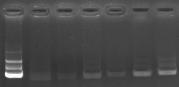

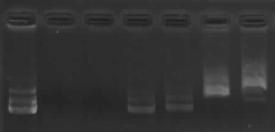


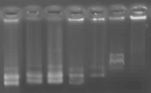

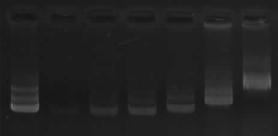


(a) (b)

Fig. S3. Agarose gel electrophoresis protection and release assay for CS/pDNA complexes prepared by cationic starch with different molecular weight (the up Mw is 1.792×10^4^ and the bottom is 5.527×10^4^) at different weight ratios (from left to right is naked DNA, 3, 6, 10, 20, 40, 60 and cationic starch respectively) under different pH ((a) pH4.0; (b)pH7.4)

Fig. S4. Hydrodynamic size (Z-average) and zeta potential of CS/pDNA nanocomplexes in PBS (pH=7.4).

Fig. S5. pH dependent size distribution curve of CS/pDNA prepared at w/w=55.
